# Salivary Fistule

**Published:** 1871-03

**Authors:** 


### ARTICLE VII.

### *Salivary Fistule.*

Here is a young woman who has been troubled with open ulcer upon her cheek for nearly two years. Her difficulty commenced as an alveolar abscess, from the second molar tooth, a disease the varied results of which I so often bring before you; (and do you not remember last summer, how I then urged upon you the necessity of a thorough comprehension of the subject, since you would so often meet with its victims?) This abscess opened not into the mouth, but burrowed upward through the buccinator muscle, perforated the

3

Stenonian duct, and finally opened upon the cheek, forming a fistulous track which communicated not only with the root of the tooth, but also with this duct, and we consequently have both saliva and pus con stantly exuding from this orifice. Here, then, are two indications to be fulfilled in the treatment. In the first place the primary cause must be removed, and this cause exists in the two remaining carious fangs of the tooth above mentioned, which will continue to exercise their irritating influence as foreign bodies so long as they remain in their present position. They must, therefore, be removed, thus giving nature the power to complete the removal of the difficulty. In the second place, having removed the cause of drainage, the saliva must be turned into its normal receptacle, the mouth.

This ulcer has resisted various treatments during these two years, for although its salivary nature was recognized, yet the underlying causes, these carious fangs, were not removed, and of course the discharge continued and must have an outlet. As these roots are below the margin of the gum, we shall use the "elevator" for their extraction. [Tooth roots here lifted out by the "elevator."]

Now we have removed the exciting cause of all this difficulty, and will next try to cure this intractable ulcer upon the cheek. What must we do? We must make a passage by which the saliva will find a more easy outlet, thus turning it out of its present channel and giving opportunity for repair. For the accomplishment of this purpose a strand of silk is threaded at each end to a straight or curved needle, and these needles are successfully passed into the fistula and carried out through the mouth, leaving about a line of tissue between the two points of passage. Removing the needle, a loose knot is then tied, forming a short loop, or else the intervening tissue may be immediately strangulated, and the loop allowed to ulcerate its way through. As this separation occurs, the saliva will take its course through this opening into the mouth (provided it has been made of sufficient size), and the external wound will heal usually of its own accord, but may sometimes require slightly stimulating with arg. nitr. I think that you will seldom be required to pare the edges of the external wound. This operation I prefer to that of Horner, where the whole tissue is cut out with a sharp saddler's punch, as you will find described in your surgical works, since it is perfectly simple and reliable.

Another mode of operation is by the use of a conical cotton tent, which is inserted into the wound after a puncture has been made with a bistoury entirely through into the mouth, its base being placed in that portion of the track situated nearest the inside of the cheek, with the delicate point of the pyramid at the external fistule. This, by its unequal expansion, will dilate the internal orifice and permit the narrowing of the external, when, after a few days, it may be removed and a similarly shaped cone of folded iron-wire inserted in its plaee, the apex of which cone should consist of but a single wire. This will induce a patulous condition of the oral passage, while the fistule will diminish in diameter to the size of the wire, when you will have but to remove it, and all will we well in two days.

Some one of these operations will usually be found applicable, but in cases where the fistule is the result of extensive sloughs, as in cancrum oris, autoplasty may be required.

These salivary fistules you will find to baffle all your attempts by stimulants and caustics, and I would rather advise you to operate at once. The worst cases are those where some of the lobules of the parotid glands have been injured; and let me here caution you to be extremely careful, in all your operations in the region of the gland, not to cut through that strong fascia, for should you cut but one lobule of the gland, a fistule may result.

[The two needles were then carried through, bearing the thread with them; a loop was made and allowed to hang loose in the mouth. In a few days it had cut a passage into the mouth; the saliva followed its tract, and after a single application of arg. nitr. the girl reported in two weeks entirely well.—De F. W.]—*Professor Garretson's Clinic. Medical and Surgical Reporter.*

